# Integrating transcriptomics and network analysis-based multiplexed drug repurposing to screen drug candidates for M2 macrophage-associated castration-resistant prostate cancer bone metastases

**DOI:** 10.3389/fimmu.2022.989972

**Published:** 2022-10-26

**Authors:** Jinyuan Chang, Zhenglong Jiang, Tianyu Ma, Jie Li, Jiayang Chen, Peizhi Ye, Li Feng

**Affiliations:** National Cancer Center/National Clinical Research Center for Cancer/Cancer Hospital, Chinese Academy of Medical Sciences and Peking Union Medical College, Beijing, China

**Keywords:** drug repurposing, castration-resistant prostate cancer, bone metastases, network pharmacology, M2 macrophage

## Abstract

Metastatic castration-resistant prostate cancer (CRPC) has long been considered to be associated with patient mortality. Among metastatic organs, bone is the most common metastatic site, with more than 90% of advanced patients developing bone metastases (BMs) before 24 months of death. Although patients were recommended to use bone-targeted drugs represented by bisphosphonates to treat BMs of CRPC, there was no significant improvement in patient survival. In addition, the use of immunotherapy and androgen deprivation therapy is limited due to the immunosuppressed state and resistance to antiandrogen agents in patients with bone metastases. Therefore, it is still essential to develop a safe and effective therapeutic schedule for CRPC patients with BMs. To this end, we propose a multiplex drug repurposing scheme targeting differences in patient immune cell composition. The identified drug candidates were ranked from the perspective of M2 macrophages by integrating transcriptome and network-based analysis. Meanwhile, computational chemistry and clinical trials were used to generate a comprehensive drug candidate list for the BMs of CRPC by drug redundancy structure filtering. In addition to docetaxel, which has been approved for clinical trials, the list includes norethindrone, testosterone, menthol and foretinib. This study provides a new scheme for BMs of CRPC from the perspective of M2 macrophages. It is undeniable that this multiplex drug repurposing scheme specifically for immune cell-related bone metastases can be used for drug screening of any immune-related disease, helping clinicians find promising therapeutic schedules more quickly, and providing reference information for drug R&D and clinical trials.

## Introduction

It is undeniable that prostate cancer has become the second leading cause of death in men (1). Prostate cancer is characterized by hormone responsiveness, and androgen deprivation therapy can make tumor regression in prostate cancer patients (2). However, most patients progress to castration-resistant prostate cancer (CRPC) after a period of castration therapy, and 85% of patients with prostate cancer develop bone metastases (BMs) and are resistant to immunotherapy (3–5). To date, bone metastases remain an incurable form of prostate cancer with a significant impact on disease-specific morbidity and mortality (5), and represent a major challenge for advanced fatal prostate cancer.

Tumor-associated macrophages (TAMs) in the microenvironment have been proven to account for more than 50% of the tumor mass and are key drivers of tumor progression, metastasis and therapeutic resistance. M1-like TAMs with antitumor effects and M2-like TAMs with protumor effects coexist within the microenvironment, and the opposing effects of these M1/M2 subsets on tumors directly affect current strategies for antitumor immunotherapy. In addition, macrophages exhibit dynamic plasticity in the tumor microenvironment and can transform from an antitumor M1-like phenotype to an M2-like phenotype during certain specific immune responses, thus exerting a tumor-supporting influence (6). Studies have shown that macrophage infiltration is associated with poor prognosis in non-small cell lung cancer, hepatocellular carcinoma, pancreatic ductal adenocarcinoma (PDAC), glioblastoma, and bladder cancer (7). Stimulated by colony-stimulating factor, it increases the risk of BMs of lung cancer and breast cancer (8). In addition, osteoclasts formed by their differentiation are involved in bone remodeling, repair and homeostasis regulation, and are considered to be one of the driving factors of tumor BMs. Inhibiting or depleting macrophage infiltration in the bone microenvironment can effectively prevent BMs. Although CRPC patients with BMs also have features of immunosuppression, differences in macrophage phenotypes have rarely been reported in such patients, and it is unclear whether macrophages in the bone microenvironment are associated with the BMs of CRPC.

In terms of therapeutic drugs, the currently approved bone-targeted drugs, monoclonal antibodies (denosumab), and radiopharmaceuticals provide some benefits, effectively reducing bone pain and pathological fractures in patients with BMs of prostate cancer, and improving the overall quality of life in these patients (9, 10). However, a large proportion of patients still experience skeletal-related events (SREs) during treatment, and safety and tolerability issues often need to be considered. Adverse effects, represented by nephrotoxicity and severe hypocalcemia, usually limit the long-term use of drugs for BMs (11). In addition, immunotherapy, which plays a role in most solid tumors, showed dissatisfactory efficacy in patients with BMs, suggesting a state of immunosuppression in these patients (5). Therefore, the scientific community needs to identify, test and approve new therapeutic compounds targeting the specific relatively immunosuppressive bone microenvironment of patients with BMs to improve the symptoms of BMs in CRPC patients, overcome adverse drug reactions, and prolong patient survival.

However, drug research and development (R&D) is usually an energy-intensive, low-yield process. Therefore, prioritization of promising therapeutic drugs based on preclinical evaluation of pharmacoinformatics and repurposing of existing drugs are often worthwhile and necessary (12, 13). With the accumulation of available data, a variety of preclinical drug R&D methods have been proposed to assist researchers in making informed decisions. Computational chemistry-based ligand-receptor binding conformational modeling has been widely used in pharmacodynamics and pharmacokinetics studies, and has played a crucial role in understanding and identifying drug-target interactions (14–16), providing a method for micromechanics analysis in the complex stable system formed by small molecules and targets (17–19). For example, the study of the interaction between anthocyanins and human serum albumin transferrin complexes using spectral, calorimetric, stopped flow and molecular modeling approaches provides a new perspective for elucidating the cyclic distribution of anthocyanins (20). Here, by integrating transcriptomic and drug susceptibility data, and based on network analysis, a multiplex drug repurposing scheme was used to investigate and comprehensively evaluate the immune infiltration landscape, differentially expressed genes (DEGs) mediating immune infiltration-related BMs, and promising candidate drugs in CRPC patients with BMs. To provide usable information for drug R&D and repurposing targeting DEGs of M2 macrophage-related BMs.

## Methods

### Patients and datasets

The microarray datasets GSE32269 (including 22 tumor samples and 29 BM samples) and GSE77930 (including 22 tumor samples and 20 BM samples) with clinical information were downloaded from Gene Expression Omnibus (GEO) (https://www.ncbi.nlm.nih.gov/gds) (21), and used as the discovery set and validation set, respectively. Specifically, the GEOquerry (22) package of R was used to download data, the hgu133a.db package of R was used to convert gene probe ID into gene symbol, and the Normalized between Arrays function in the limma (23) package of R was used for data normalization.

### DEGs analysis and functional enrichment analysis

Genes with differential expression between the BM group and the primary group in the GSE32269 and GSE77930 cohorts were analyzed using the R package limma. And adjusted p< 0.05, and |log2FoldChange| > 1 were used as filter conditions. Functional enrichment analysis was performed using the R package clusterProfiler, and Gene Ontology (GO) and Kyoto Encyclopedia of Genes (KEGG) terms with adj p<0.05 were considered significant.

### Transcriptome-based assessment of immune infiltration

The immune score, stromal score and tumor purity were calculated for each tumor sample in the primary and BM groups, using the ESTIMATE algorithm. Based on the ssGSEA method, the tumor immune microenvironment signatures of primary and BMs were inferred using a manually curated gene expression signature of 29 immune microenvironment functional genes by Alexander Bagaev et al. (24). The content of infiltrated immune cells in the tumor microenvironment of the primary and BM groups was calculated using the EPIC and Timer methods encapsulated by the R package IOBR and the CIBERSORT method provided online (25–27). The EPIC and Timer methods were executed with default parameters. For the CIBERSORT method, gene expression profiles prepared from standard annotation files were uploaded to the CIBERSORTx web portal (https://cibersortx.stanford.edu/) and run using the LM22 gene signature file and 1,000 permutations. To ensure the accuracy of the results, only samples with a CIBERSORT p value< 0.05 were retained for further analysis, and immune cells whose content was 0 in more than half of the samples were excluded. Immune cells with statistical significance and similar infiltration patterns in more than two algorithms were considered reliable.

### Evaluation of DEGs and pathways in M2 macrophage-associated BMs

A random forest classifier was constructed using the randomForest package to identify the genes most associated with the BM phenotype of CRPC, ranking in importance according to the mean decrease accuracy value (28). Then, 5 times of ten-fold cross-validation were performed, and the number of important genes was selected according to the cross-validation curve. Permutation tests were performed on important genes using the rfPermute package, and significance information for each gene was obtained. Gene set enrichment analysis (GSEA) was performed with the R package Pi to explore the upregulation of pathways in the BMs group of CRPC (29). Specifically, the HALLMARK gene set was downloaded for quantification of pathway activity. The GSEA algorithm was run with 10,000 permutations using the gene list sorted by Log2FC as input, followed by the Benjamini-Hochberg method to control for FDR. Pathways with gene peaks greater than 30 and FDRs less than 0.05 were considered significantly enriched. Between each method, protein-protein interactions (PPI) based on the STRING database were used to screen for DEGs associated with M2 macrophages in BMs (30).

### Transcriptome-based multiplex drug repurposing

The obtained prostate cancer BMs differential genes were input into the Connectivity Map (31), L1000CDS^2^ (32) and L1000FWD (33) tools, respectively. Since the output of L1000CDS2 was limited to 50 drugs, the same cutoff was chosen for other databases, and the databases were sorted according to their reverse enrichment scores (inhibition scores). The drug scores from three different datasets were calculated with reference to the method proposed by the researcher Marios Tomazou to normalize the ranking of drugs using the weighting of the average ranking and the number of occurrences, which were used as input for the prior score of CoDReS. In this study, the weights of each part of CoDReS are defined as waS=0.45, wFS=0.45, and wStS=0.1 (34, 35).

Transcriptome-based repurposed drug structures were searched and downloaded, entered into the ChemBioServer 2.0 tool, used to calculate distance matrices for chemical and structural similarity, and clustered the drugs using the Ward method with a minimum Tanimoto similarity of 80% (36). The drug with the highest ranking according to the CoDReS normalized score in each cluster was selected to eliminate redundant structures in the drug list.

### Transcriptome-based drug sensitivity analysis

The R package oncoPredict was used to assess the sensitivity of CRPC patients with BMs to chemotherapeutic drugs (37). The package was based on a ridge regression model that used expression data and drug response data from cancer cell lines to train the model to predict drug sensitivity from a patient’s gene expression data. Drug response data for human cancer cell lines were obtained from Genomics of Drug Sensitivity in Cancer (GDSC1&2, https://www.cancerrxgene.org/), and expression data for GDSC1&2 cancer cell lines were obtained from the GDSC1000 resource (https://www.cancerrxgene.org/gdsc1000/). Drugs with NA values in more than 20% of cell lines were discarded. The k-nearest neighbors (KNN) method was used to estimate the remaining missing values.

### Network-based natural compound screening

In the HERB database, the cell or tissue type was set to be derived from prostate cancer to screen the natural compounds, and the obtained compounds were used as the keyword input in the “Differentially expressed genes” module to obtain the potential action target of the compound (38). Cytoscape 3.7.2 was used to construct a natural compound-prostate cancer BMs network, calculating the criticality of natural compounds in the network according to the formula (1), and normalizing the ranking of key natural compounds within the unit interval (–1, 1) by dividing by the absolute maximum score. Key compounds were classified by structure, and extensive virtual screening of compounds in the same category was performed in the MedChemExpress library. ADMETLAB 2.0 was used to comprehensively evaluate the pharmacophysicochemical properties and pharmacokinetics of candidate compounds in the natural compound database, and molecules with reasonable conformations and low toxicities were considered as promising inhibitors (39).


$$ScoreBM_{i}=\frac{DegreeBM_{reverse_{i}}-DegreeBM_{mimic_{i}}}{N_{S_{i}\cap S_{BM}}\times \text{max}\left|ScoreBM\right|}$$

$$ScoreM2_{i}=\frac{DegreeM2_{reverse_{i}}-DegreeM2_{mimic_{i}}}{N_{S_{i}\cap S_{GSEA}}\times \text{max}\left|ScoreBM\right|}$$

i=1,...,N Drugs

(1) $$DR_{i}=\omega_{BM}\times ScoreBM_{i}+\omega_{M2}\times ScoreM2_{i}$$

*Degree_reverse_* indicates that natural compounds regulate transcription in the reverse direction (with antagonistic effects) to BMs differential genes. *Degree_mimic_* indicates that natural compounds regulate transcription in the same direction (with synergistic effects) as BMs differential genes. *S_i_* represents the gene set related to natural compounds. *S_GSEA_* represents the gene set generated by GSEA. In this study, *ω_BM_* =0.3 and *ω_M2_* =0.7 were set respectively.

### Molecular docking and virtual screening

The 3D structure of the compound was downloaded, and if only 2D structures were available, chem3D was used to draw the 3D structure and optimize the force field. The structural information of key targets was retrieved and predicted through the PDB database and AlphaFold Protein Structure Database, respectively. The most potential ligand binding sites were found based on the cocrystals, protein cavities and literature reports. The Arg-Gly-Asp (RGD) structure of SPP1 was the main site where it is bound by receptors and mediates signaling. The RGD polypeptide structure of SPP1 was obtained from a cocrystal of 1L5G (PDBID) (40).

The protein and compound structures were imported into AutoDock software. The compound was set to be flexible and the center coordinates were set according to the ligand binding site. The Lamarckian genetic algorithm was used to evaluate the binding ability between the ligand and the protein (41).

### Allosteric sites of SPP1 receptor proteins based on D3pocket and DCC (dynamic cross-correlation matrices) analysis

The SPP1 receptor structure downloaded from PDB was used as input to the D3pocket and R package Bio3D tools (42, 43). The orthosteric and allosteric sites were represented using PyMOL in light blue and red, respectively. DCC was used to analyze the trajectory after Gromacs dynamics simulation.

### Molecular dynamics

Gromacs was used for 10 ns molecular dynamics simulations of the candidate compounds and to perform an ensemble equilibration of temperature and pressure at 310 K and 1 Bar, followed by positional confinement of proteins and small molecules, respectively (44, 45). The last frame structure after simulation equilibrium was used as the input of the allotype to predict the potential function of candidate compounds (46).

### Comparison with ongoing clinical trials

Clinical studies related to castration-resistant prostate cancer bone metastases were obtained from ClinicalTrials.gov. Using “Prostate Cancer”, “Castration resistant”, and “Bone Metastasis” as keywords, the structures of small molecule drugs and drugs reported for clinical research were obtained from PubChem. All candidate compounds were further used as input to Chembioserver 2.0.

## Result

### Transcriptome-based DEGs analysis

**Figure 1:**
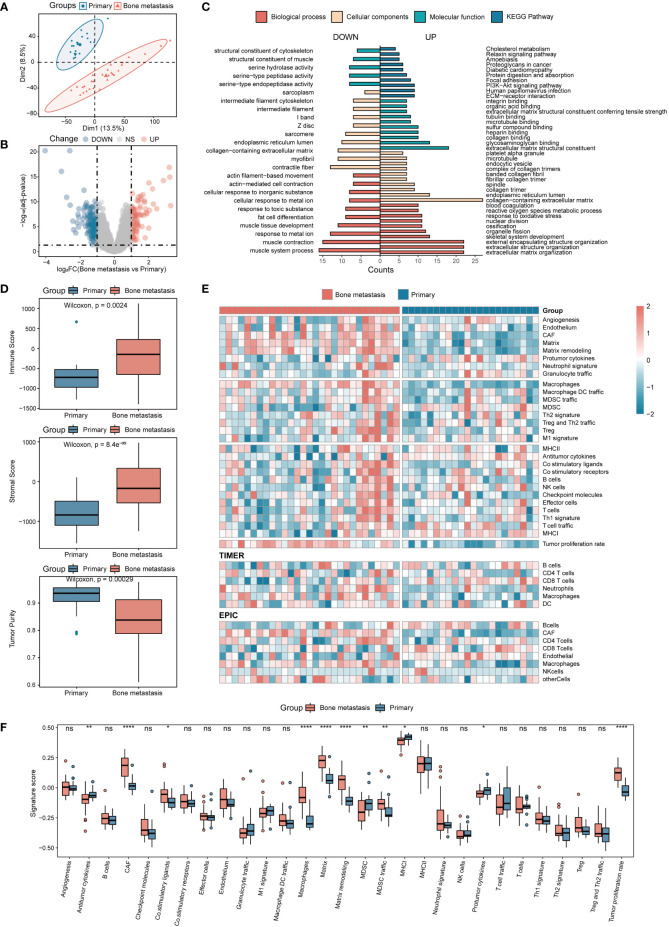
Analysis of DEGs and immune infiltration in CRPC with BMs. **(A)** Sample principal component analysis. **(B)** Analysis of DEGs in BMs. **(C)** DEGs of BMs enrichment analysis. **(D)** Immune, stromal, and tumor purity scores in patients between the primary focus and BMs. **(E)** Immune infiltration analysis. **(F)** Twenty-nine immune cell characteristics in patients with BMs. *P<0.05; **P<0.01; ****P<0.0001. ns, not significant.

To detect the dispersion between samples, PCA was conducted on the included microarray data. As shown in **Figures 1A**, **S1D**, samples can be clearly divided into two categories, indicating that the samples have good intragroup consistency and intergroup heterogeneity. By analyzing the differences in transcriptome expression between the two groups of patients, in the discovery set, a total of 229 genes with significant differences were finally obtained, of which 89 were upregulated and 140 were downregulated (**Figure 1B**). The biological process of differential genes was mainly enriched in the formation of extracellular matrix and extracellular structure, which was positively correlated with the maintenance of extracellular structure, and was involved in the signal transduction of integrin binding, cell adhesion and extracellular matrix receptor interaction pathway (**Figure 1C**).

Immune infiltration was further used to analyze the mechanism of BMs from the perspective of immune cell composition. The results showed that compared with the primary samples, the microenvironment of BMs contained more immune cells and stromal cells, and the tumor purity was relatively low (**Figure 1D**). Further analysis of immune cell composition revealed that based on the SSGSEA tool, a total of 11 significant changes in immune infiltration components were obtained. Among them, immune cells represented by macrophages and tumor-associated fibroblasts were significantly increased in BMs, while antitumor cytokines and MHC-I were significantly reduced (**Figures 1E, F**). Based on the EPIC and TIMER tools, four and two different abundances of infiltrating immune cells were obtained, respectively. Among them, the abundance of CD8+ T cells, macrophages and tumor-associated fibroblasts in BMs were significantly higher than that in the primary focus (**Figures 1E**, **S1A, B**). Based on the CIBERSORT tool, a total of 2 different types of immune cells were obtained, including activated NK cells elevated in the primary focus and M0 macrophages elevated in BMs. Meanwhile, in the discovery set, M2 macrophages, resting NK cells, and T regulatory cells were also enriched in BMs (**Figure S1C**). Interestingly, the validation set and the discovery set were highly consistent in the immune cell infiltration results, suggesting a certain degree of reliability and reproducibility of the above results (**Figures S1F-H**). The results of immune infiltration showed that BMs were enriched in macrophages and deficient in CD8+ T cells, and the results were mutually validated by more than two approaches in both the discovery set and the validation set. Due to the important role of macrophages in bone homeostasis, this study focused on the further exploration of their involvement in BMs from the perspective of macrophages.

**Figure 2:**
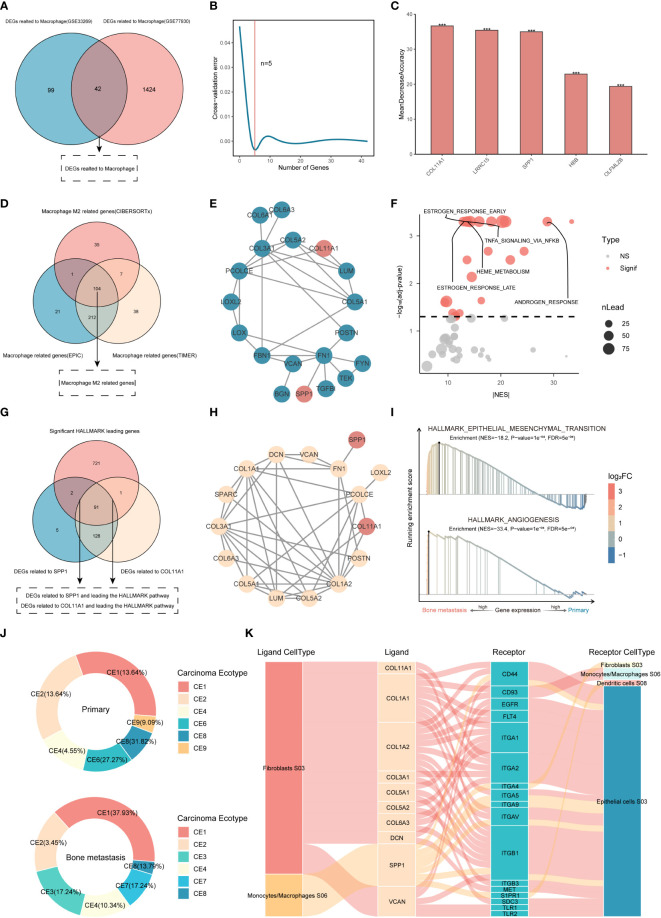
Identification of genes characteristic of M2 macrophage-associated BMs. **(A)** Macrophage-related DEGs. **(B, C)** Key gene identification based on random forest. ***P<0.001. ns, not significant. **(D)** Analysis of M2 macrophage-related DEGs based on WGCNA. **(E)** PPI between DEGs of BMs and M2 macrophage-related genes. **(F)** GSEA of SPP1 and COL11A1 proteins. **(G, H)** PPI analysis between proteins in enrichment pathways and key proteins. **(I)** GSEA of kernel targets. **(J, K)** Cancer ecotype analysis and cellular distribution of ligands and receptors.

The DEGs obtained by the TIMER and EPIC methods were correlated with macrophage phenotypes. As shown in **Figure 2A**, under the condition that the correlation is greater than 0.3 and is significant, 141 and 1466 macrophage-related DEGs were obtained in the discovery set and the validation set, respectively, of which a panel of 42 DEGs was simultaneously proven to be related to macrophage phenotype by two methods in the two datasets. The random forest method was further used to identify the genes with the ability to distinguish BMs in this collection. When the threshold was set to 5 (**Figure 2B**), the genes represented by COL11A1 were obtained, and the set constituted by them had the maximum discrimination ability (**Figure 2C**).

Generally, macrophages are divided into two subtypes, M1 and M2, with different biological functions. It is necessary to explore which subtypes are enriched in BMs. Although the content of M2 macrophages did not show differences in the validation set, the content of M2 macrophages in BMs showed an upward trend (**Figure S1H**), while it was significantly increased in the discovery set. Therefore, based on the WGCNA method, this study further explored the DEGs associated with M2 macrophages in the discovery set. Simultaneously, the correlation between each gene module and the abundance of macrophages obtained using the EPIC and TIMER methods was calculated based on the Pearson correlation coefficient. Finally, 699 genes related to macrophages (338 based on the EPIC method and 361 based on the TIMER method) and 147 genes related to M2 macrophage were extracted (**Figures S2, S3**). To further confirm the M2 macrophage-related genes, this study used the macrophage-related genes derived from the EPIC and TIMER methods as a universal set, including but not limited to the M0, M1 and M2 macrophage subtypes, and further intersected them with the related genes of M2 macrophages obtained by CIBERSORT analysis. A total of 104 genes with the potential to regulate M2 macrophages were identified based on WGCNA of the three immune infiltration algorithms (**Figure 2D**), suggesting that these genes play a crucial role in regulating the phenotype of M2 macrophages in CRPC.

To further explore the relevant genes that can predict BMs and participate in direct or indirect regulation of the M2 macrophage phenotype, 104 M2-related genes, 10 M2 macrophage markers (http://xteam.xbio.top/CellMarker/), and 5 macrophage-related genes with the ability to differentiate bone metastasis were used for PPI analysis, and the results showed that SPP1 and COL11A1 were considered to be the key DEGs with both the ability to differentiate between BMs and to regulate M2-macrophages (**Figure 2E**).

GSEA was further used to analyze genes related to SPP1 and COL11A1, and 22 pathways closely related to the BMs of CRPC were screened (**Figure 2F**). The enriched DEGs in the pathway were 93 and 92, with the ability to regulate BMs, related to SPP1 and COL11A1, respectively (**Figure 2G**). Interestingly, both had identical PPI networks under the set threshold, and the obtained set of 16 genes played a more central role in M2 macrophage-mediated BMs (**Figure 2H**), which were used as kernel inputs for subsequent drug repurposing studies. Functionally, it was mainly enriched in the pathways of epithelial-mesenchymal transition and angiogenesis (**Figure 2I**).

The results of cancer ecotype analysis showed that there were significant differences in the ecological composition. Compared with the primary focus, patients with BMs accounted for more CE1 and CE4, but less CE8, and had specific CE3 subtypes and lacked CE6 and CE9 subtypes. The 16 kernel genes represented by SPP1 and COL11A1 were mainly distributed in two ecological subtypes, CE1 rich in macrophages and CE3 rich in fibroblasts and epithelial cells (**Figure 2J**). Proteins represented by integrin, CD44 and S1PR1 distributed in fibroblasts, epithelial cells and dendritic cells were considered to be receptors for kernel genes (**Figure 2K**).

### Drug sensitivity analysis

**Figure 3:**
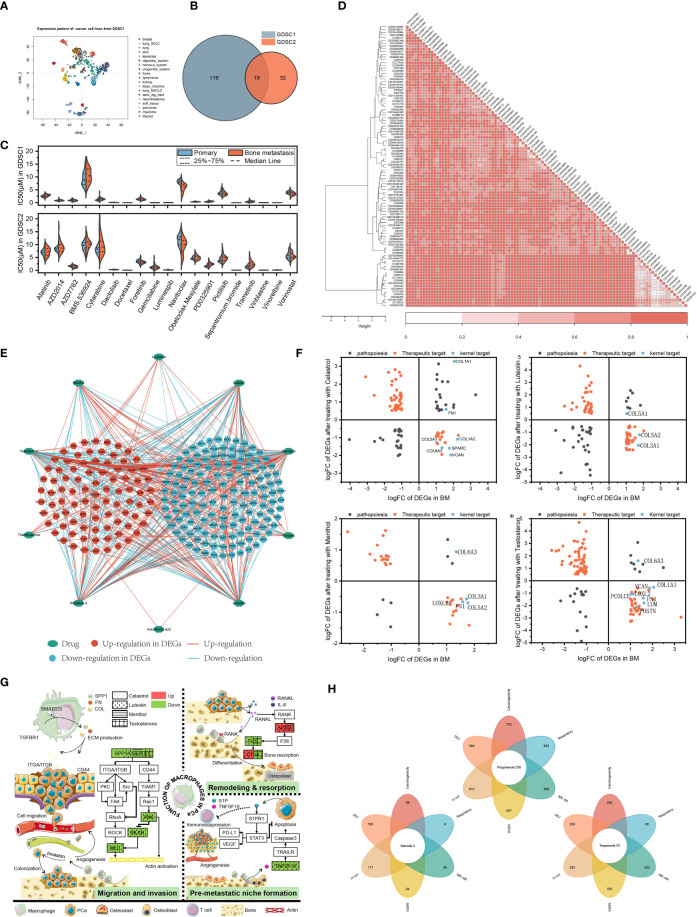
Integrating transcriptomes and network-based drug repurposing. **(A)** Cellular gene expression signature. **(B)** Sensitive drugs with co-occurrence in GDSC1&2. **(C)** IC50 of drugs in primary focus and BMs. **(D)** Structure-based drug cluster analysis. **(E)** Network pharmacology of natural compounds. **(F)** Topology-based candidate natural compounds. **(G)** Pathway patterns of candidate natural compounds. **(H)** Rational structure compounds in the MedChemExpress database.

Hundreds of cancer cell line gene expression data and drug response data from GDSC1&2 were used to train a ridge regression model to infer the susceptibility of patients in primary focus versus BMs to different drugs. Since cancer cell lines in the blood system have different gene expression signatures from most other cancer cell lines (**Figure 3A**), they were excluded to ensure the reliability of the predicted results. Drugs with IC50 values less than 10 μM and repeated in GDSC1&2 were considered as potential drugs for the treatment of prostate cancer BMs. The results showed that GDSC1&2 included 138 and 51 BMs-sensitive drugs that met the screening conditions, respectively, of which 19 co-occurring drugs were repeatedly verified by two databases to have anti-CRPC and BMs potential (**Figure 3B**).

Further IC50 studies of the drugs showed that compared with primary focus, navitoclax was more sensitive to BMs (7.65 μM) and the efficacy was consistent in the GDSC1&2 databases (P<0.05). Patients were more sensitive to drugs represented by docetaxel (mean IC50 of 0.00857 μM and 0.0114 μM in patients with primary focus and BMs, respectively) and sepantronium bromide (mean IC50 of 0.0129 μM and 0.0155 μM, respectively) (**Figure 3C**).

### Transcriptome-based multiplex drug repurposing

The transcriptome-based drug collection was sorted and normalized, and a total of 102 structurally-specific drugs with the potential to negatively regulate DEGs in the BMs of CRPC were obtained (**Table S1**). Hierarchical clustering analysis revealed that the input drugs spanned a broad diversity of chemical structures. Specifically, 38 clusters were obtained, of which 31 contained more than one drug (**Figure 3D**). By calculating the normalized CoDReS scores of the drugs, a total of 38 drugs represented by taxifolin were finally obtained. Combined with GDSC, 19 drugs with potential sensitivity to BMs were used as positive controls for the subsequent natural compound screening.

### Screening of natural compounds based on network topology

According to the screening conditions, 10 natural compounds with potential regulation of prostate cancer were obtained. According to the constructed network, among the obtained natural compounds, testosterone had the most intersecting genes with a total of 134, followed by Withaferin A and Celastrol (**Figure 3E**). To standardize and normalize the number and regulation direction of DEGs contained in natural compounds, the obtained natural compounds were further calculated according to formula (1) in this study. Menthol, testosterone, luteolin and celastrol had higher scores and potential therapeutic effects on the BMs of prostate cancer (**Table S2**). **Figure 3F** also showed that more of the 16 kernel targets obtained by GSEA fall into regions with therapeutic potential. Although Withaferin A had more intersecting genes, it was excluded from subsequent studies due to its undesirable logFC value in GSEA-related targets.

To further clarify the role of natural compounds in macrophage-related BMs, the four natural compounds obtained in formula (1) were subjected to enrichment analysis, and a potential pathway map was drawn. As shown in **Figure 3G**, all four could regulate the expression of collagen or SPP1, thereby exerting regulatory effects on the invasion and metastasis of prostate cancer cells. Meanwhile, menthol, testosterone and luteolin could also regulate the differentiation of osteoclasts, participating in the regulation of bone remodeling and resorption balance. CTSK protein, as one of the specific markers of osteoclasts, testosterone and luteolin have diametrically opposite regulatory directions. As an androgen, testosterone can inhibit bone resorption, enhancing bone strength, which is the same as the potential function of androgen. Luteolin can inhibit the secretion of TNFSF10 from macrophages, regulating the formation of the pre-metastatic microenvironment.

Based on the assumption of structural similarity and functional similarity, 380 terpenoids, 124 steroids and 881 polyphenols in MedChemExpress were included for further investigation. ADMETLAB 2.0 was used to evaluate the structural plausibility of the included natural compounds. Among them, polyphenols, terpenoids and steroids had a total of 236, 33 and 3 candidate compounds that met the criteria, respectively (**Figure 3H**).

### Mulberroside C and terrestrosin D have higher affinity

**Figure 4:**
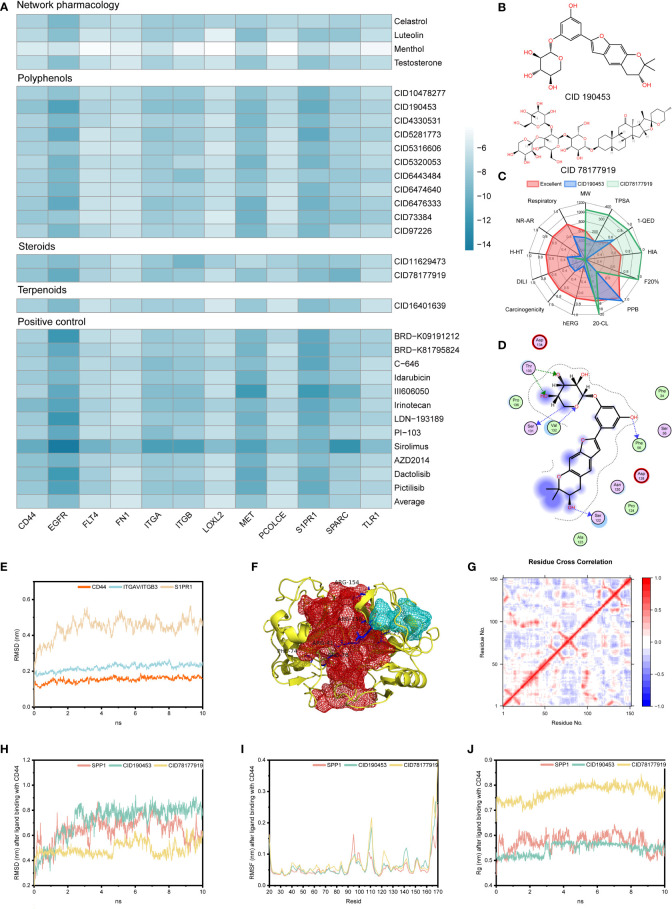
Stability evaluation of candidate compounds. **(A)** Docking simulation of candidate compounds. **(B)** Chemical formulas of CID 190453 and CID 78177919. **(C)** Evaluation of pharmacophysicochemical properties based on ADMETLAB2.0. **(D)** The docking pattern of CID 190453 with CD44. **(E)** Molecular dynamics simulation of SPP1 receptors. **(F)** Allosteric (red pocket) and orthosteric sites (blue pocket) of CD44. **(G)** DCC analysis of CD44. **(H-J)**. RMSD, RMSF and Rg analysis of candidate compounds after binding to the SPP1 receptor.

Molecular docking was performed on the candidate natural compounds, and the positive drugs obtained in the above process were used as controls (**Figure 4A** and **Table S3**). Two compounds were obtained (**Figure 4B**), CID 190453 (mulberroside C) and CID 78177919 (terrestrosin D), both of which had a higher affinity to the targets than the average value of the positive control. Determining the binding mechanism depends on fundamental thermodynamic parameters, such as binding free energy, which can be calculated from hydrogen bonds formed between ligands and proteins, electrostatic forces, van der Waals forces, and hydrophobic interactions (47, 48). To further quantify the binding ability of the ligand to the protein, this study further predicted the binding constant by AutoDock (**Table 1**), suggesting that the binding constants of mulberroside C and terrestrosin D to the receptors of SPP1 were both at the nanomolar level, showing good spontaneous binding ability.

**Table 1:** Binding energies and binding constants of mulberroside C and terrestrosin D to the receptors of SPP1.

Target name	Compound name	Binding energy	Ki [Temperature = 298.15 K] (nM)
CD44	CID 190453	-8.5	589.94
	CID 78177919	-8.4	698.31
ITGAV/ITGB3	CID 190453	-8.8	355.72
	CID 78177919	-8.6	498.40
S1PR1	CID 190453	-10.3	28.35
	CID 78177919	-9	253.88

**Figure 4C** showed that both have good safety profiles, among which mulberroside C has relatively excellent oral absorption and drug metabolism. Although terrestrosin D has poor oral absorption, which is the same as steroid drugs, it has the better plasma protein binding (PPB) and distribution ability, suggesting that mulberroside C can be administered orally, and terrestrosin D can be administered intravenously or intramuscularly.

Both of them can not only bind to CD44, ITGAV/ITGB3 and S1PR1 receptors through hydrophobic forces, but also form hydrogen bonds to improve the binding stability (**Figures 4D**, **S4**). In addition, the compounds occupied the residue site of SPP1 interacting with these three receptors, which affected the normal function of the signaling pathway.

### Stability and activity analysis of mulberroside C and terrestrosin D based on dynamics and allotype tools

Dynamics simulations of CD44, ITGAV/ITGB3 and S1PR1 were performed using Gromacs, and the equilibrated trajectory files were used as input to D3Pocket and bio3D (**Figure 4E**).

Previous studies showed that hyaluronate binds to the far N-terminal domain of CD44 (the red pocket) and does not affect the OPN-CD44 interaction (**Figure 4F**). Throughout the trajectory, the dynamic correlation of residue sites where the red pocket was located showed a negative correlation with residues within the blue pocket (**Figure 4G**). Here, blue and red pockets were used to bind SPP1 and natural compounds, respectively. Similarly, as shown in **Figures S5A–D**, the red pocket residues of ITGAV/ITGB3 and S1PR1 exhibited a dynamic correlation with the residues in the blue pocket and were further investigated as allosteric and orthosteric sites, respectively.

Molecular dynamics studies of small molecules and receptors showed that in 10 ns simulations, both SPP1 and candidate compounds reached equilibrium, fluctuating root mean square deviation (RMSD) values over time compared to the position of the CD44 receptor pocket, fluctuating between 0.015 and 0.656 nm, with similar volatility (**Figure 4H**). Compared with the CD44 receptor, the RMSD fluctuation of candidate compound binding to the ITGAV/ITGB3 receptor was more stable. However, mulberroside C did not stably bind to the allosteric pocket of S1PR1 (**Figures S5E, F**).

In addition, after binding of mulberroside C and terrestrosin D, the fluctuation of residues had different peaks than those of SPP1 (**Figures S5G–I**). Taking CD44 as an example, compared with the binding of SPP1, the flexibility of receptor residues 40-60 was higher after binding to the candidate compound, and reached the peak around residues 111 and 165, while these residues showed lower flexibility when binding to SPP1. Similarly, after binding SPP1, CD44 was significantly more volatile at residue 95 than the allosteric site-binding candidate compound (**Figure 4I**). This may be related to the ligands occupying the receptor pocket, which affected the flexibility of the residue by forming an interaction force, thereby affecting the movement of the residue at the orthosteric site by binding to the allosteric site, and then hindering the function of proteins.

The rigidness of the compound in the system can be addressed through the inspection of the radius of gyration (Rg) value. As shown in **Figures 4J**, **S5J, K**, after the candidate compounds bound to CD44, the Rg remained at approximately 0.546 ± 0.023 nm and 0.771 ± 0.029 nm, and the SPP1 fluctuates around 0.572 ± 0.032 nm. Among them, the Rg of terrestrosin D was significantly larger than that of mulberroside C and SPP1, which may be related to its complex structure.

The last frame after 10 ns simulation was used as the input file of the allotype tool to predict the function after the receptor binds to the allosteric pocket. The results show that the ΔΔG values of the candidate compounds are all negative (**Table 2**), suggesting that the function of the candidate compounds is to inhibit the binding of the protein to SPP1, thereby inhibiting the pathway.

**Table 2:** Calculated ΔΔG Values for candidate compounds after binding to the SPP1 receptors using AlloType.

Protein	PDBID	Ligand	Predicted allsoteric type	ΔΔG (kcal/mol)
CD44	4PZ3	CID 190453	Inhibition	32.5070
		CID 78177919	Inhibition	41.7766
ITGAV/ITGB3	1L5G	CID 190453	Inhibition	39.1615
		CID 78177919	Inhibition	35.5584
S1PR1	3V2Y	CID 78177919	Inhibition	11.5344

### Evaluation of the integrated drug list with respect to ongoing clinical trials

Cluster analysis was performed on 62 drugs and clinical trials obtained from multiplex drug repurposing, and a total of 48 clusters were obtained, of which 2 drugs (docetaxel and sirolimus) were already in clinical trials. Eight structurally specific drugs (danazol, 3-Cl-AHPC, 5-fluorocytosine, rilmenidine, BRD-K09191212, SB-225002, PD-0325901, and obatoclax mesylate) were also obtained. Among them, PD-0325901 was reported to have BMs sensitivity in the GDSC database.

In the remaining clusters, structurally similar drugs to current clinical trials were highlighted (**Figure S5L**), and the most promising repurposing drugs that could play a role in the treatment of BMs of CRPC by interfering with M2 macrophages were further screened according to the following three principles: (a) drugs that were similar to phase 3 or 4 clinical trials and belonged to the same cluster, (b) drugs that have the ability to regulate the M2 macrophage-associated BMs genes obtained by modulating GSEA, and (c) drugs that have been reported in the literature to have prostate cancer therapeutic potential.

The criteria were met by 5 drugs on the list (norethindrone, testosterone, docetaxel, menthol, and foretinib). Among them, testosterone and docetaxel have been used in phase 2 and phase 3 clinical trials, respectively. Although experts still have concerns about the use of testosterone in prostate cancer, there have been several phase II clinical trials investigating “bipolar androgen therapy (BAT)” for CRPC (49). The five drugs mentioned above have the potential to further become clinical drugs for BMs of CRPC.

## Discussion

The median overall survival in metastatic CRPC was only 13 months (50), underscoring the need for treatment. Immunotherapy has been successfully used in the treatment of a variety of tumors, however, accumulating evidence suggests that prostate cancer is a “cold” immune desert with low immune infiltration, low tumor mutational burden, and low antigen presentation. Therefore, prostate cancer does not respond as strongly to a single immune checkpoint inhibitor treatment as it does for immune “hot” tumors represented by non-small cell lung cancer, which leads to limited response to immunotherapy and suggests the immunosuppressive state of patients with BMs (51). Due to the dynamic balance between “osteoblasts” and “osteoclasts” inherent in bone, bone has a relatively unique immune microenvironment. Studies have shown that in the tissue samples of patients with osteolytic metastasis of prostate cancer, an increase in immune infiltration represented by macrophages and T cells was observed. Compared with osteolytic metastasis, the content of macrophages in osteogenic bone metastasis was significantly reduced. In addition, the immune checkpoint B7-H3 is upregulated in tissue samples from patients with BMs, suggesting that prostate cancer BMs have immunogenic characteristics distinct from those of the primary tumor (52). In particular, immune cells represented by macrophages not only play an important role in bone homeostasis, but also participate in the regulation of bone formation (11). Studies have shown that the number of M2 macrophages and the activity of inflammasomes were positively correlated with bone tumor burden (10).

The results of this study also showed that BMs of CRPC have higher immune scores and more macrophages than the primary focus, but the number of CD8+ T cells (EPIC & TIMER) and activated NK cells (CIBERSORT) in BMs is lower, with more M2 macrophages, T regulatory cells (CIBERSORT) and tumor related fibroblasts (ssGSEA & EPIC). Moreover, the abundance of MHC I-related antigen-presenting molecules (ssGSEA) was lower than that in the primary focus, which resulted in BMs with relatively low immunogenicity. Thus, the absence of such “cytotoxic” cells and the infiltration of “immune response suppressor” cells makes the microenvironment of CRPC patients with BMs more closely resemble those of cold immune tumors. Although BMs have significantly higher immune scores than primary tumors, immune checkpoint therapy for patients with BMs has not been successful. This is related to the unique composition of T cell populations and the infiltration of immunosuppressive cells in patients with BMs (5, 53). These findings underscore the importance of careful assessment of immune infiltration in CRPC patients with BMs to guide drug use.

Simultaneously, a collection of DEGs identified from CRPC with BMs highlighted in enrichment analysis extracellular matrix and integrin-related pathways that were strongly associated with prostate cancer metastasis. Specifically, among the DEGs, a total of 141 genes were involved in the regulation of macrophages. Through random forest, WGCNA, GSEA and PPI, it was finally determined that SPP1 and COL11A1 were related to M2 macrophages with the ability to predict BMs.

As a highly specific osteolysis biomarker, SPP1 and type I collagen have been previously shown to be expressed and secreted by a variety of cancers, and participate in cell adhesion, bone resorption, cell adhesion, metastasis and other processes by binding to CD44 and integrin receptors (54–58). As a major mediator of tumor-associated inflammation, SPP1 has been proven to be related to enzalutamide resistance by activating the PI3K/AKT and ERK1/2 signaling pathways in CRPC, and promoting the invasion and metastasis of CRPC (59). COL11A1 was shown to be involved in immune-related pathways and was significantly associated with RFS in patients (60). In consideration of their important roles in CRPC, both have been suggested by investigators as alternative prognostic assessments and new promising immunotherapy targets for drug development.

Based on the DEGs of the above two groups of patients, integrated transcriptomic and network-based analysis combined with existing clinical trials to screen promising drugs for repurposing, a total of 5 nonrepetitive drugs were obtained (norethindrone, testosterone, docetaxel, menthol, and foretinib), and should receive special attention.

As a progesterone derivative, norethindrone inhibits 5α-reductase, a key protease that converts testosterone to dihydrotestosterone, has been proven to reduce bone mineral loss in male castrated mice, and has been used in the treatment of hormone-refractory prostate cancer (61). Here, this study highlights its bone-protective effect through the M2 macrophages, which can be further used in the prevention and treatment of CRPC bone metastases.

Different from progesterone, testosterone, as an important androgen, has a role in promoting the occurrence and growth of prostate cancer. Although studies have shown that testosterone levels correlate with disease progression, and that androgen deprivation therapy can lead to prostate cancer tumor regression (2), patients inevitably enter a castration-resistant stage, where castration-resistant therapy is no longer effective. Studies have shown that in prostate cancer, the Gleason score is negatively correlated with testosterone dependence, and highly aggressive prostate cancer does not depend on testosterone. Artificial supplementation with exogenous testosterone can inhibit the further progression of such highly aggressive prostate cancer, thereby reducing prostate cancer invasion risk (62, 63). Several clinical trials have been conducted using BAT for CRPC. Considering the important role of testosterone in bone health, if exogenous testosterone supplementation is no longer a contraindication for CRPC, we have reason to believe that the application of testosterone will be a promising treatment for BMs of CRPC.

Docetaxel, a drug that has been clinically approved for CRPC treatment, has been shown to prolong the survival of prostate cancer patients with more than 4 BMs (64). It also shows that the drugs obtained based on integrated transcriptomic and network-based analysis have certain robustness and reproducibility.

As a multiple receptor tyrosine kinase inhibitor, foretinib exhibited potent inhibition of c-MET, vascular endothelial growth factor receptor 2 (KDR) and FLT4, and showed antitumor and antiangiogenic activities. High expression of c-MET was found in 83% of prostate cancer BMs, and inhibitors targeting this protein have been used in clinical trials at various stages (65).

As a terpenoid, menthol can bind to TRPM8 and has been approved for the treatment of bronchitis and rhinitis. TRPM8, as a member of the transient potential receptor family, has been shown to be highly expressed in androgen-sensitive cancer cells, is a potential prognostic marker for metastatic CRPC, and is also considered a promising druggability target for the treatment of prostate cancer (66). Although prostate cancer cells depend on the Ca^2+^ infiltration of TRPM8 for invasion and metastasis, non-physiological activation of TRPM8 by menthol inhibits the proliferation and motility of CRPC (67, 68). Screening potent specific agonists for activating TRPM8 channels will be one of the strategies for future drug R&D.

Natural compounds are considered a treasure trove of drug discovery, with an estimated 25-38% of innovative FDA-approved chemical drugs derived from phytochemicals or their derivatives (69). Network-based and integration of existing natural compound transcriptome sequencing results of prostate cancer cells. Two of the four potential drug candidates (Menthol and Testosterone) were included in the final candidate list, showing the referential role of network pharmacology in drug R&D. Through further analysis of the natural compound database, two potential compounds were finally obtained, namely, mulberroside C and terrestrosin D. Both have greater affinities for receptor proteins than the average positive drugs in the virtual screening. In addition, in the follow-up molecular dynamics, except that mulberroside C and S1PR1 failed to bind stably, they all showed good stability in the 10 ns simulation.

Allostery is a phenomenon in proteins where functional changes in the active site result from distant perturbations (such as ligand binding and mutation). In general, allosteric can be analyzed as a thermodynamic energy cycle, and it is usually necessary to predict the allosteric ability of drugs before R&D. In 2021, Professor Lai’s research group from Peking University proposed a tool called allotype to predict the direction of allosteric regulation based on the force distribution in the binding site, which is used to calculate the allosteric coupling strength ΔΔG (46). The results of Allotype also showed that both have the ability to inhibit the binding of SPP1 to the receptor, thereby inhibiting the activation of downstream pathways. In terms of inhibitory ability, compared with terrestrosin D, which has a greater inhibitory ability against CD44, mulberroside C has a stronger inhibitory ability against ITGAV/ITGB3. Among the three receptors, the inhibitory ability of CD44 and ITGAV/ITGB3 was stronger, but that of S1PR1 was weaker. However, this study was mainly based on theoretical calculations, and the binding energy of the drugs were not measured experimentally, which may result in a discrepancy between the two. The main reasons for the difference may be as follows (70): (a) the sampling strategies and scoring criteria for Lamarck genetic algorithms and grid calculations used in the molecular docking process limit the increase in accuracy. (b) The molecular weight of the ligand is too large or contains multiple polar groups, which participate in the formation of various electrostatic interactions. (c) Insufficient sampling of ligand parameters such as spatial position, orientation, distance and conformation resulted in the failure to fully consider the effect of the internal energy contained in the candidate compounds on the binding energy during the docking process. Despite the problem of false positives or false negatives during virtual screening, hit quality improves with the number of compounds screened (71). A total of 1686 compounds were included in this study, and 328 small molecules were evaluated by molecular docking simulation. The ligand pose after binding of protein was dynamically evaluated by molecular docking and molecular dynamics. It is possible to eliminate false positives or false negatives for binding energies caused by incorrect ligand posture. Although they were not included in the list of the most promising repurposed drugs through the final screening conditions, there were also experimental studies showing that terrestrosin D has the effect of inhibiting the growth of prostate cancer and anti-angiogenesis (72).

Drug repurposing is used to rapidly identify and develop therapeutics for unmet needs. However, the plasma concentrations of many newly discovered compounds are lower than the required drug concentrations, limiting their direct clinical use (73). Combinations in tumor therapy, originally proposed to overcome drug resistance and provide new treatment options (74), have been used as a way to increase the success rate of drug repurposing. Two drugs that exhibit synergistic effects in clinical treatment allow the drug to achieve the same level of efficacy as a high-dose single drug at a lower dose, thereby reducing the dose of one drug and improving clinical safety. Tumor pathogenesis usually involves pathological features characterized by redundancy and versatility, limiting the clinical efficacy of single-target drugs. However, drug combination therapy often results in complex pharmacodynamic or pharmacokinetic interactions, or both, due to individual differences and other factors, which makes it difficult to describe the effectiveness and side effects of combined drugs, and may bring additional health issues (75). The evaluation of drug absorption, distribution, metabolism, excretion and toxicity characteristics is of great significance for predicting drug interactions. Most antitumor drugs need to undergo extensive liver metabolism, such as drugs metabolized by microsomal cytochrome P-450. When other drugs used in combination inhibit the activity of these enzymes, it is easy to cause drug interactions *in vivo* and affect drug efficacy. In this study, through the multiplex drug repurposing method, among the five candidate compounds obtained, docetaxel combined with abiraterone was used for the first-line treatment of metastatic CRPC, with a lower rate of serious adverse events (76). Although the phase II clinical study of foretinib showed that all patients included experienced at least one adverse event (77), its combination with PD-1 has been shown to be applicable in the treatment of colorectal cancer (78), suggesting the value of candidate compounds in combined medication.

Naturally, this work still has certain limitations. At present, only two transcriptome dataset has been included for DEGs analysis, and were used as the discovery set and validation set. The results may have potential bias, which still needs the support and proof of a quantity of transcriptome data in the future. In addition, the transcriptome sequencing results of natural compounds against prostate cancer cells are limited. Although this study simulated the natural compound database with molecular docking and dynamics, the real situation may still differ from the simulation. Finally, we used a multiplex-drug repurposing approach integrating transcriptomes and network-based approaches to generate a drug candidate list. Although these drugs have demonstrated clinical or experimental antitumor effects, for bone metastases, the primary site of treatment is equally important, which requires consideration of the toxic and adverse reactions of combined pharmacotherapy in clinical use, and evaluation of drug safety. Here, the research based on the method of pharmacoinformatics provides new insights for the repurposing of drugs that are already in the experiment, exploring the new indications of drugs from the perspective of synthesis and prediction, and provides a new scheme for the treatment for BMs of CRPC.

## Data availability statement

The datasets presented in this study can be found in online repositories. The names of the repository/repositories and accession number(s) can be found in the article/**Supplementary Material**.

## Author contributions

LF is the corresponding author of this article, JCa and ZJ contributed equal. LF conceived the research. JCa collected the data and drafted the manuscript. ZJ analyzed the data and TM and JL provided valuable suggestions on the investigation. JCe and PY critically reviewed the manuscript and assisted in the final write-up of the manuscript. All authors contributed to the article and approved the submitted version.

## Funding

This research was supported by the cancer discipline construction project of integrated traditional Chinese and Western medicine of Peking Union Medical College (20192010302), the Beijing Natural Science Foundation (7222293) and the special subject of research and evaluation of China Association of Chinese Medicine (CACMRE2021-A-05).

## Acknowledgments

In this study, we sincerely thank Mr. Zixuan Chai from the Cancer Hospital Affiliated to Chongqing University for his guidance and help in the process of bioinformatics analysis.

## Conflict of interest

The authors declare that the research was conducted in the absence of any commercial or financial relationships that could be construed as a potential conflict of interest.

## Publisher’s note

All claims expressed in this article are solely those of the authors and do not necessarily represent those of their affiliated organizations, or those of the publisher, the editors and the reviewers. Any product that may be evaluated in this article, or claim that may be made by its manufacturer, is not guaranteed or endorsed by the publisher.
