# Supplementary material for: Integrating transcriptomics and network analysis-based multiplexed drug repurposing to screen drug candidates for M2 macrophage-associated castration-resistant prostate cancer bone metastases
Source: Front Immunol. 2022 Oct 26;13:989972. doi: 10.3389/fimmu.2022.989972 (PMC9643318; doi:10.3389/fimmu.2022.989972)
Supplement: Supplementary file 1 [file DataSheet_1.docx]

**Supporting Information**

Integrating transcriptomics and network analysis-based multiplexed drug repurposing to screen drug candidates for M2 macrophage-associated castration-resistant prostate cancer bone metastases

Jinyuan Chang^a#^, Zhenglong Jiang^a#^, Tianyu Ma^a^, Jie Li^a^, Jiayang Chen^a^, Peizhi Ye^a^, Li Feng^a*^

^a^ National Cancer Center/National Clinical Research Center for Cancer/Cancer Hospital, Chinese Academy of Medical Sciences and Peking Union Medical College, Beijing (100021), China

# Contributed equally

* Correspondence: fengli663@126.com; Tel.: +86-010-877-880-30

**Validation of differential expression and immune infiltration analysis of castration-resistant prostate cancer bone metastases**

Through the discovery set, based on EPIC and TIMER, 4 and 3 different abundances of immune infiltrating cells were obtained, respectively. Among them, the abundance of CD8+ T cells, macrophages and tumor-associated fibroblasts in BMs was significantly increased (Figure S1A-B). Based on CIBERSORT, a total of 6 kinds of immune cells with different contents were obtained, including resting DC cells and activated NK cells, which were increased in the primary focus, but M0 and M2 macrophages, resting NK cells and T regulatory cells, which were increased in bone metastasis (Figure S1C).

The analysis results of the validation set showed that the samples could be clearly divided into two categories, indicating that the samples had good intragroup consistency and intergroup heterogeneity (Figure S1D). There were 3826 differentially expressed genes in total, of which there were 1960 and 1866 upregulated and downregulated differentially expressed genes, respectively (Figure S1E).

The samples were further analyzed for immune infiltration. Interestingly, in the samples included in the GSE77930 dataset, the immune infiltration results obtained based on both the EPIC and TIMER methods were consistent with the discovery set in significance and content (Figure S1F-G). Compared with the discovery set, in the validation set, the content of DC cells (TIMER) in BMs was higher than that in the primary tumor, while the content of B cells and CD4+ T cells was slightly lower (TIMER). Based on the CIBERSORT, the results of M0 macrophages and activated NK cells in the discovery set and the validation set were consistent. Although the content of M2 macrophages did not show differences in the validation set, it can be seen from Figure S1H that in BMs, the content of M2 macrophages showed an upward trend, and the insignificant difference may be related to insufficient sample size.

The correlations of 229 and 3826 differentially expressed genes obtained from the discovery set and the validation set with macrophages were used to further explore the differentially expressed genes that have the ability to differentiate BMs and regulate the phenotype of macrophages. The results showed that 141 and 1466 differentially expressed genes played a role in the discovery set and the validation set, respectively (Figure S1I-J).

**Module eigengenes associated with macrophages in the microenvironment of castration-resistant prostate cancer bone metastases**

Macrophage-related genes in the CRPC were identified by weighted co-expression network analysis (WGCNA) using the WGCNA package of R software. The top 5000 genes with the highest MAD values were extracted to construct the gene co-expression network. β = 6 (scale-free R2 = 0.87) or β = 5 (scale-free R2 = 0.86) were chosen as soft threshold parameters to ensure a scale-free network (Figure S2 A–B). Then, we calculated the Pearson correlation coefficient between each pair of extracted genes to generate an adjacency matrix. The adjacency matrix was subsequently transformed into topological overlap measure (TOM) by using the tomlikeity function. Using the average linkage hierarchical clustering algorithm based on the TOM-based dissimilarity measure, genes with similar expression patterns were clustered into a same module. In addition, 15 and 29 gene modules, respectively, were found in the two weighted co-expression networks, and the number of genes in each module varied from 43 to 1021 (Figure S2 C–D). Next, to acquire the modules related to immune cells, we used Pearson correlation analysis to calculate the correlation between each immune cell and the module eigengenes (MEs), and further calculated the gene significance (GS), module membership (MM) and module significance (MS). MEs are the main component and the most representative expression mode of each gene module. The GS represents the correlation between gene expression and immune cells. The MM is defined as the correlation between gene expression values and the MEs. The MS is the average GS value of all genes in the module. The results showed that yellow modules (based on the EPIC and TIMER methods) and pink modules (based on CIBERSORT methods) were acquired for subsequent analysis (cor > 0.1, p < 0.05) (Figure S2G).

Among them, the correlation coefficient between the yellow gene module and macrophages was 0.79 (Figure S3A-D), and the correlation coefficient with the pink module was 0.65 (Figure S3E-F). Finally, 699 genes related to macrophages (338 based on the EPIC method and 361 based on the TIMER method) and 147 genes related to M2 macrophage were extracted. It is suggested that these genes play a crucial role in regulating the phenotype of CRPC.

**Docking simulation of mulberroside C and terrestrosin D**

After virtual screening, studies have shown that mulberroside C and terrestrosin D have strong affinity with SPP1 receptors, and both can not only bind to CD44, ITGAV/ITGB3 and S1PR1 receptors through hydrophobic forces, mulberroside C (Figure S4A-B) can also form hydrogen bonds with ITGAV/ITGB3 (at SerB123, TyrB166 and ArgB214) and S1PR1 (at Lys34, Ser105 and Val194), respectively, and terrestrosin D (Figure S4C-E) can also form hydrogen bonds with CD44 (at Arg29, Phe56, Asn57 and Asp128), ITGAV/ITGB3 (at AlaA215, AspA218, SerB121, ArgB214, AsnB215, AlaB218 and AsnB313) and S1PR1 (at Ser3, Asp40, Asn101, Ser105, Gly106, Thr109 , Glu121, Val194, Arg292 and Glu294) to increase the stability of the binding. Meanwhile, the candidate compounds occupy exactly the residue sites where SPP1 interacts with these three receptors, hindering the interaction of SPP1 with the receptors. In combination with ITGAV/ITGB3 and S1PR1, mulberroside C occupied the SerB123 and ArgB214 residues of ITGAV/ITGB3 and Lys34 of S1PR1, respectively, and terrestrosin D occupied the ASPA218, SERB121, ASNB215 residues of ITGAV/ITGB3 and Asp40 and Gly106 of S1PR1, respectively, hindering the formation of hydrogen bonds between SPP1 and receptors, affecting the normal function of SPP1 (Figure S4F-H).

**Allosteric regulatory potential of mulberroside C and terrestrosin D**

Red pocket residues (allosteric sites) of ITGAV/ITGB3 and S1PR1 exhibit dynamic correlations with residues within blue pockets (orthosteric sites). Among them, the allosteric sites of ITGAV/ITGB3 were mainly composed of VAL51, ALA43, GLU15, and GLN180, and the orthosteric sites were mainly composed of ASP150, SER121, and ASP218 (Figure S5A). The above residues were negatively correlated in the DCC analysis (Figure S5B). LEU276 plays a critical role in the allosteric site of the S1PR1 receptor, showing a positive correlation in DCC with ARG120 and ARG292 residues that constitute the orthosteric site (Figure S5C–D).

The candidate compounds were docked into the protein receptor pockets, and the RMSD of the candidate compounds fluctuated more stably after binding to the ITGAV/ITGB3 receptor than that of the CD44 receptor in the 10 ns simulation. After binding to the ITGAV/ITGB3 receptor, the fluctuation of mulberroside C gradually increased within 0-2 ns, finally reached the 0.5 nm position at 2 ns, and then maintained equilibrium. After binding to the ITGAV/ITGB3 receptor, terrestrosin D briefly increased during 0–1.5 ns, followed by a sustained decrease, and reached equilibrium at approximately 0.33 nm at 4 ns (Figure S5E). Different from the previous two receptors, after binding to the allosteric pocket of S1PR1, mulberroside C fluctuated around 0.35 ns within 0-5 ns, gradually increased after 5 ns, and finally left the binding pocket at 8.8 ns. In the 10 ns dynamics simulation, there is no stable binding to the allosteric site. Terrestrosin D fluctuated gradually within 0-1 ns, and after 1 ns, the trajectory reached equilibrium at 0.35 nm (Figure S5F).

The root mean square fluctuation (RMSF) of the residues was calculated to further explore the fluctuation of the backbone atoms of the candidate compound-receptor complex. The RMSF of candidate compounds after binding to ITGAV/ITGB3 was also significantly different from that of SPP1. Mulberroside C showed lower activity at residue 30 after binding to ITGAV, and terrestrosin D showed higher flexibility at residues 48, 216 and 322. However, after binding to SPP1, the flexibility at residues 353 and 382 was higher. ITGB3 showed higher flexibility when combined with terrestrosin D, while SPP1 was in the middle, and the flexibility when combined with mulberroside C was the worst (Figure S5G-H). Since mulberroside C was released from the receptor pocket of S1PR1 at 8.8 ns, the volatility of protein residues with stable binding during 5 ns was selected for study. The results showed that, compared with binding to SPP1, the flexibility of the receptor residues was enhanced after binding the candidate compound, and it was more obvious at sites 50, 75, 285-300, 1035 and 1072-1161 (Figure S5I). The Rg studies showed that the radius of gyration of terrestrosin D was significantly larger than that of mulberroside C and SPP1 after the candidate compound binds to ITGAV/ITGB3 and S1PR1 receptors, which may be related to its complex structural formula (Figure S5J-K).

**Further screening of drugs based on the ongoing clinical trials**

Based on transcriptome, GDSC and network pharmacology, a total of 11 drugs (mefexamide, oxymetholone, norethindrone, exemestane, 1-benzylimidazole, mirtazapine, BJM-ctd2-10, docetaxel, foretinib, testosterone and menthol) were obtained. These 11 drugs have certain structural similarities with the drugs in the current phase 3 and phase 4 clinical trials (Figure S5L).





Figure S1. Validation of differential expression and immune infiltration analysis of CRPC BMs. A, B and C. Immune infiltration analysis based on EPIC, TIMER, and CIBERSORT in the discovery set. *, P<0.05. **, P<0.01. ***, P<0.001. ****, P<0.0001. ns, not significant. D. Sample principal component analysis. E. Analysis of DEGs in BMs. F, G and H. Immune infiltration analysis based on EPIC, TIMER, and CIBERSORT in the validation set. *, P<0.05. **, P<0.01. ***, P<0.001. ****, P<0.0001. ns, not significant. I and J. Macrophage-related DEGs based on EPIC and TIMER, in the discovery and validation sets, respectively.





Figure S2. Identification of module eigengenes associated with macrophages of CRPC by WGCNA. A and B. Network topologies at various soft thresholds. C and D. Linkage hierarchical clustering of 5,000 modular eigengenes. E, F and G. Heatmaps of correlations between gene modules and macrophages, based on the EPIC, TIMER and CIBERSORTx methods.


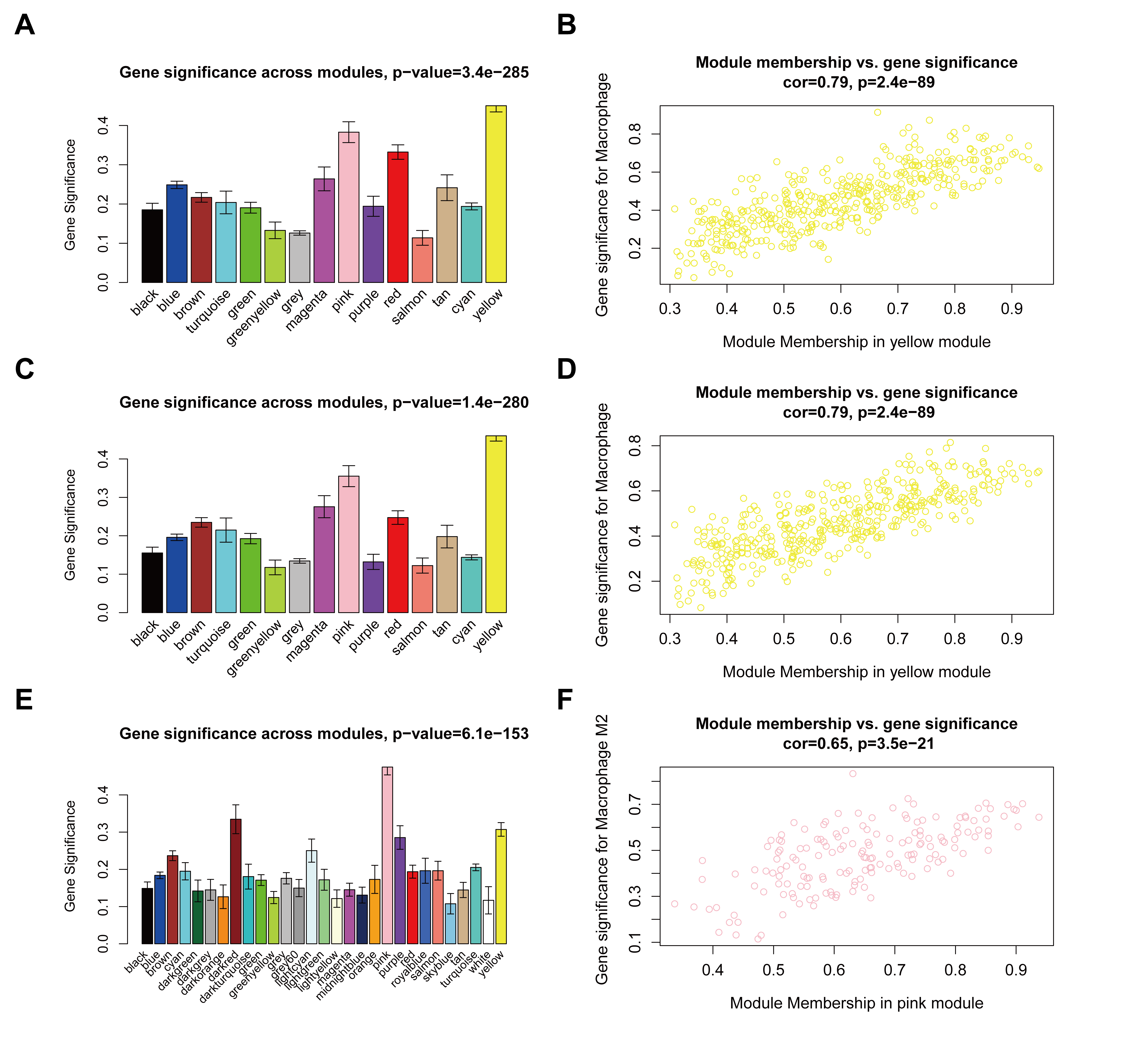


Figure S3. Module and phenotype association analysis. (A,C and E) Distribution of mean gene significance and errors in macrophage-related modules. Based on EPIC (A), TIMER (C) and CIBERSORT (E) respectively. (B, D and F) Intramodule analysis to identify macrophage-related genes with high module membership and gene significance. Based on EPIC (B), TIMER (D) and CIBERSORT (F) respectively.


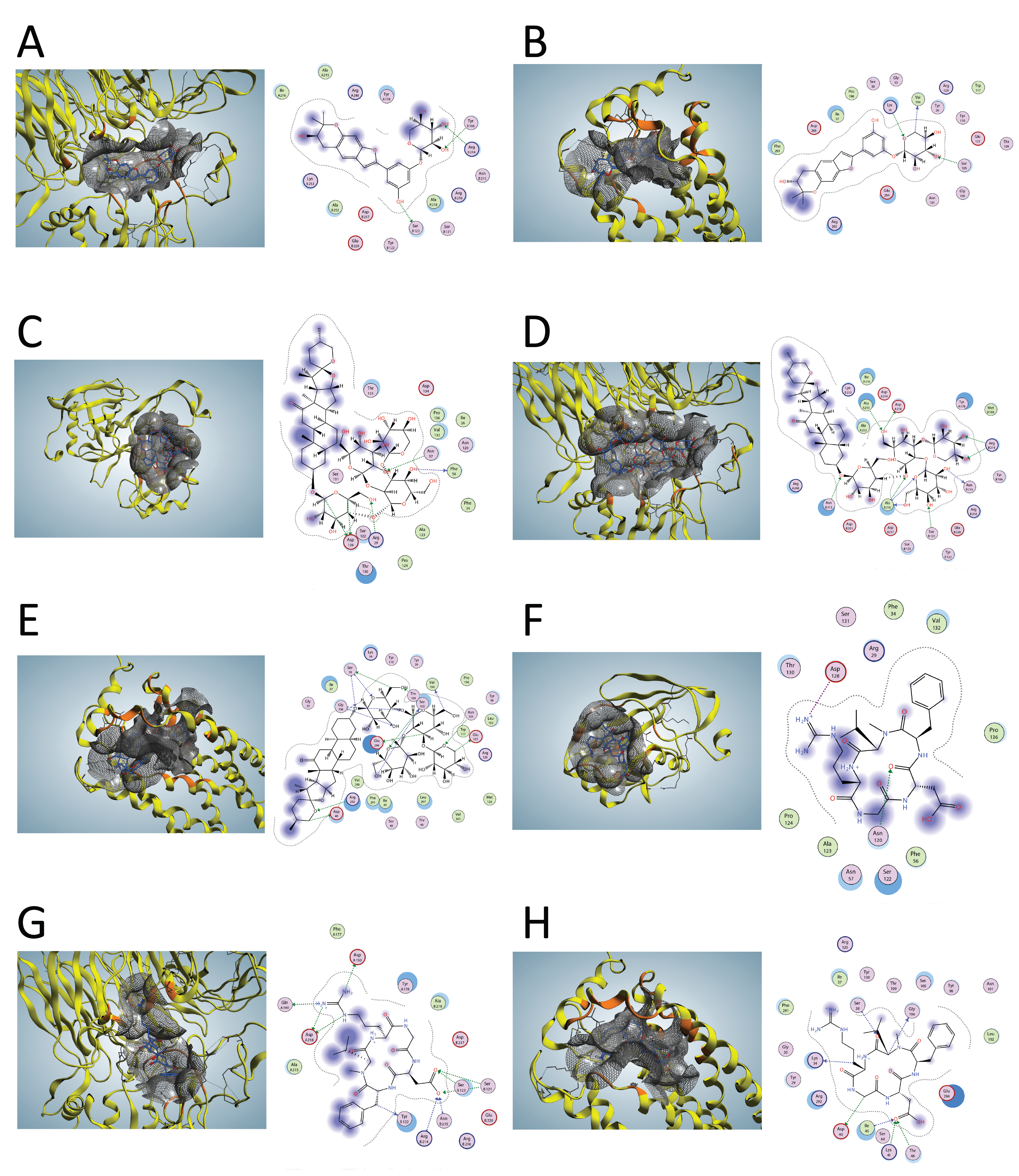


Figure S4. Interaction pattern of candidate compound binding to receptors. A-B. Interaction pattern of mulberroside C with ITGAV/ITGB3 and S1PR1. C-E. Interaction pattern of terrestrosin D with CD44, ITGAV/ITGB3 and S1PR1. F-H. Interaction pattern of SPP1 with CD44, ITGAV/ITGB3 and S1PR1.


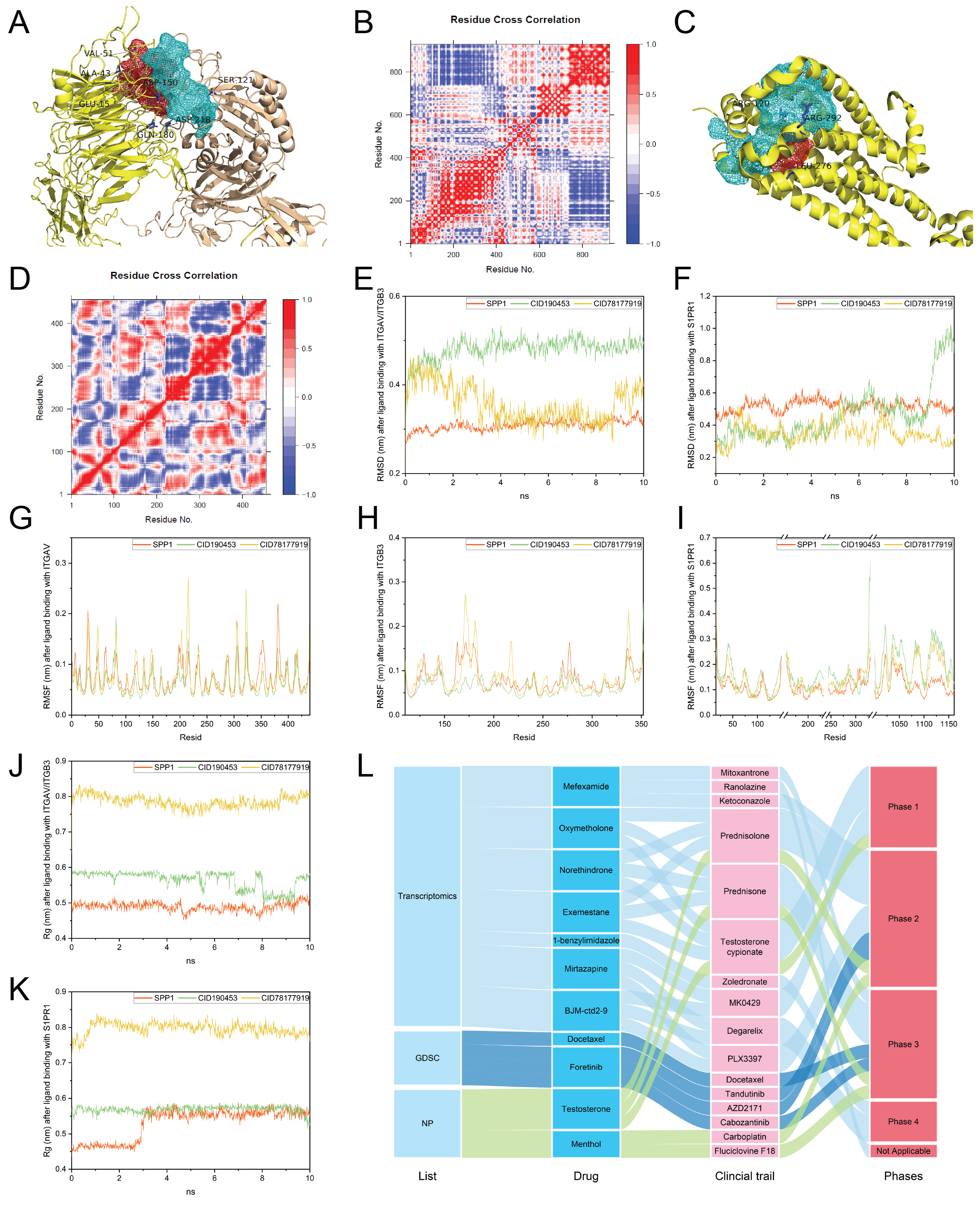


Figure S5. Molecular dynamics-based stability studies of candidate compounds. A and C. Allosteric (red pocket) and orthosteric sites (blue pocket) of ITGAV/ITGB3 and S1PR1. B and D. DCC analysis of ITGAV/ITGB3 and S1PR1. E-F. RMSD analysis of candidate compounds after binding to the ITGAV/ITGB3 and S1PR1. G-I. RMSF analysis of candidate compounds after binding to the ITGAV/ITGB3 (G and H) and S1PR1 (I). J-K. Rg analysis of candidate compounds after binding to the ITGAV/ITGB3 and S1PR1. L. Clinical trial-based drug candidate screening.
